# No association between *IFNL3 (IL28B)* genotype and response to peginterferon alfa-2a in HBeAg-positive or -negative chronic hepatitis B

**DOI:** 10.1371/journal.pone.0199198

**Published:** 2018-07-17

**Authors:** Lai Wei, Heiner Wedemeyer, Yun-Fan Liaw, Henry Lik-Yuen Chan, Teerha Piratvisuth, Patrick Marcellin, Jidong Jia, Deming Tan, Wan-Cheng Chow, Maurizia R. Brunetto, Moisés Diago, Selim Gurel, Viacheslav Morozov, Hua He, Yonghong Zhu, Cynthia Wat, Bernadette Surujbally, Alexander J. Thompson

**Affiliations:** 1 Peking University People’s Hospital, Peking University Hepatology Institute, Beijing, China; 2 Department of Gastroenterology, Hepatology and Endocrinology, Hannover Medical School, Hannover, Germany; 3 Liver Research Unit, Chang Gung Memorial Hospital, Chang Gung University College of Medicine, Taipei, Taiwan; 4 Department of Medicine and Therapeutics, Institute of Digestive Disease and State Key Laboratory of Digestive Disease, The Chinese University of Hong Kong, Hong Kong, China; 5 NKC Institute of Gastroenterology and Hepatology, Prince of Songkla University, Songklanagarind Hospital, Songkhla, Thailand; 6 Service d’Hépatologie and INSERM CRB3/U773, Université Paris-Diderot, Clichy, France; 7 Beijing Friendship Hospital, Capital Medical University, Beijing, China; 8 Xiangya Hospital, Central South University, Changsha, Hunan Province, China; 9 Department of Gastroenterology & Hepatology, Singapore General Hospital, Singapore, Singapore; 10 Hepatology Unit, University Hospital of Pisa, Pisa, Italy; 11 Hospital General de Valencia, Valencia, Spain; 12 Department of Gastroenterology, Medical Faculty, Uludag University, Bursa, Turkey; 13 Medical Company Hepatolog, LLC, Samara, Russian Federation; 14 Roche Products Ltd, Welwyn, United Kingdom; 15 Genentech Inc., San Francisco, California, United States of America; 16 BStats Solutions Ltd, Hertfordshire, United Kingdom; 17 St Vincent’s Hospital, University of Melbourne, Fitzroy, Victoria, Australia; Centre de Recherche en Cancerologie de Lyon, FRANCE

## Abstract

**Background & aims:**

It has yet to be firmly established whether host *IFNL3* (*IL28B*) genotype influences interferon responsiveness in patients with chronic hepatitis B. We investigated associations between single-nucleotide polymorphisms (SNPs) in the *IFNL3* region and response to peginterferon alfa-2a in 701 patients enrolled in three large, randomized, international studies.

**Methods:**

Responses were defined as hepatitis B surface antigen (HBsAg) loss and/or hepatitis B e antigen (HBeAg) seroconversion plus hepatitis B virus (HBV) DNA <2000 IU/ml in HBeAg-positive patients, and HBsAg loss and/or HBV DNA <2000 IU/ml in HBeAg-negative patients (24 weeks after end of treatment). Associations between treatment response and the number of copies of the poor-response allele at three SNPs (rs8099917, rs12980275, rs12979860) were explored with logistic regression models in Asian and white patients.

**Results:**

The HBeAg-positive and -negative populations comprised 465 (92% Asian, 50% HBV genotype C) and 236 (79% Asian, 41% HBV genotype C) patients, respectively, and had respective response rates of 26% and 47%. The *IFNL3* genotype was strongly associated with ethnicity. There was no association between *IFNL3* genotype and treatment response in HBeAg-positive or -negative patients. Independent predictors of treatment response were: sex, HBV DNA level and alanine aminotransferase level in HBeAg-positive Asian patients; age in HBeAg-negative Asian patients; and HBV DNA in HBeAg-negative white patients.

**Conclusions:**

This is the largest analysis to date of associations between *IFNL3* genotype and peginterferon response in patients with chronic hepatitis B. The data suggest that *IFNL3* polymorphism is not a major determinant of the response to peginterferon alfa-2a in either HBeAg-positive or HBeAg-negative patients.

## Introduction

Single-nucleotide polymorphisms (SNPs) in the region of the gene for interferon lambda 3 (*IFNL3*, also known as *IL28B*; Gene ID: 282617) on chromosome 19 are strongly associated with clinical outcomes in patients infected with hepatitis C virus (HCV). *IFNL3* genotype has been shown to be the strongest baseline predictor of a sustained virologic response to dual therapy with pegylated interferon (PegIFN) alfa plus ribavirin [1–6]. Differences in *IFNL3* allele frequencies between different population groups largely explain the well-established ethnic differences in response rates to interferon (IFN)-based therapies [1,7,8]. *IFNL3* genotype is also associated with the rate of spontaneous clearance of acute HCV infection [9–12]. The biological mechanism responsible for the *IFNL3* genotype associations remains poorly defined, but responsiveness to exogenous/endogenous IFN is believed to be central, reflected by the observation that patterns of intrahepatic expression of IFN-stimulated genes differ according to *IFNL3* genotype in patients with chronic HCV infection [13,14].

In contrast, it has yet to be firmly established whether, and to what extent, host *IFNL3* genotype might influence the response to IFN-based therapy in patients with chronic hepatitis B (CHB). Several retrospective analyses with discordant results have examined the association between the response to IFN-based treatment and *IFNL3* genotype [15–23]. Some studies have reported significant associations between SNPs in the *IFNL3* region and treatment outcomes in hepatitis B e antigen (HBeAg)-positive [18] and HBeAg-negative [22] patients, while other studies have reported no association between these genetic polymorphisms and treatment outcomes [15–17,19,21,23]. However, between-study comparisons have been hampered by important differences in patient populations, such as HBeAg-antigen status, ethnic background, and hepatitis B virus (HBV) genotype, as well as treatment regimens and outcome definitions. HBV genotype is particularly important. Patients infected with HBV genotypes A or B are more likely to experience HBeAg seroconversion and hepatitis B surface antigen (HBsAg) loss after treatment with IFN [24]. The prevalence of HBV genotypes varies geographically, with genotypes A and D being more prevalent in the West, and genotypes B and C being more prevalent in Asia. Thus, the present study was designed with these deficiencies in mind. We examined associations between three markers of host *IFNL3* genotype (rs12979860, rs8099917, and rs12980275) and response to 48 weeks of treatment with PegIFN alfa-2a using consistent outcome definitions in a large cohort of patients who were enrolled in three international randomized studies. The dataset included a large number of Asian and white patients with HBeAg-positive and HBeAg-negative CHB [25–27].

## Methods

### Subjects

Patients with CHB who were included in this analysis had received 48 weeks of treatment with PegIFN alfa-2a 180 μg/week with or without oral lamivudine in three large, randomized, international clinical studies [25–27]. The rationale for combining data from patients treated with and without lamivudine was that lamivudine therapy did not influence long-term outcomes with PegIFN alfa-2a in two large trials [25,26]. The complete inclusion and exclusion criteria, study designs, and primary results of these studies have been published elsewhere [25–27]; thus, only a brief overview will be provided. In the first study, HBeAg-negative patients with an HBV DNA level >100,000 copies/ml and elevated alanine aminotransferase (ALT) levels were randomized to 48 weeks of therapy with one of three treatments: (1) PegIFN alfa-2a plus oral lamivudine, (2) PegIFN alfa-2a plus matched placebo, or (3) oral lamivudine alone [25]. In the second study, the same three treatment regimens were evaluated in HBeAg-positive patients with an HBV DNA level >500,000 copies/ml and elevated ALT levels [26]. In the third study (NEPTUNE), HBeAg-positive patients with an HBV DNA level >100,000 copies/ml and elevated ALT levels were randomized to either 24 or 48 weeks of treatment with PegIFN alfa-2a at a dosage of either 90 or 180 μg/week [27]. Patients co-infected with HCV or human immunodeficiency virus and individuals with a history or evidence of decompensated cirrhosis were excluded from each of the three trials.

This pooled retrospective analysis included only patients who received PegIFN alfa-2a at a dose of 180 μg/week and for a duration of 48 weeks, had provided informed consent for *IFNL3* genotyping, and had an adequate sample available for testing.

### Sample collection, processing, and analysis

Three tag SNPs in the *IFNL3* region of chromosome 19 were investigated (rs12980275, rs8099917, and rs12979860). These SNPs were selected because they have been shown to influence the response to IFN-based therapies in patients with chronic HCV infection [1–3,9,27].

DNA was extracted from stored serum samples using the QIAamp DNA Blood Mini QIAcube Kit (Qiagen, Hilden, Germany). The concentration and presence of extracted DNA was measured and confirmed using a PicoGreen assay with a DNA/RNA spectrophotometer. Samples from China were genotyped using the MassARRAY System (Agena Bioscience, San Diego, CA, USA) at CapitalBio Corp., Beijing, China. Samples from regions outside of China were genotyped via TaqMan PCR (Roche Molecular Diagnostics, Pleasanton, CA, USA) at Quintiles-Singapore, Singapore.

### Definition of response

The pre-specified primary outcome in all three studies was treatment response at 24 weeks after the end of treatment. For the purposes of this analysis, treatment response was defined as follows: (1) HBeAg seroconversion plus HBV DNA <2000 IU/ml or HBsAg loss in HBeAg-positive patients; or (2) HBV DNA <2000 IU/ml or HBsAg loss in HBeAg-negative patients.

### Statistics

Separate statistical analyses were conducted for Asian and white patients within the HBeAg-positive and HBeAg-negative populations because of differences in HBV genotype distribution among ethnic groups. Baseline characteristics (age, sex, ethnic origin, HBV genotype, baseline ALT, baseline HBV DNA) were summarized overall and by ethnicity for the HBeAg-positive and HBeAg-negative populations. The Wald chi-square test was used to test for significant associations between treatment response and *IFNL3* genotype for each SNP. For each of the three SNPs, unadjusted Wald odds ratios, 95% confidence intervals, and associated p-values were estimated from univariate logistic regression models to assess the quantitative effect of SNP genotype on outcome, fitting the *IFNL3* SNP genotype as a fixed binary effect and treatment response as the binary outcome variable.

To identify other significant baseline predictors of treatment response, baseline covariates (sex, age, race, HBV genotype, HBV DNA level, and ALT level) were explored separately using univariate logistic regression models in HBeAg-positive Asian and white patients and in HBeAg-negative Asian and white patients. Significant associations were defined as p≤0.05. The final analysis for each SNP was an additive logistic regression model, modelling the effect of genotype, adjusted for significant baseline covariates, on treatment response. All statistical analyses were performed using SAS 9.2 (SAS Institute Inc., Cary, NC, USA).

Deviation from Hardy–Weinberg equilibrium was assessed by Fisher’s exact test, and linkage disequilibrium was assessed by the squared Pearson correlation coefficient (r^2^) for each SNP using Plink (v1.07) (http://pngu.mgh.harvard.edu/purcell/plink/) [28]. Markers yielding Fisher’s exact p-values of <0.001 were investigated for genotyping errors but were not excluded from further analysis.

### Ethics

The clinical studies included in this analysis were conducted in accordance with the Declaration of Helsinki and the laws and regulations in force in the countries in which the research was conducted. In the original studies the protocols and all amendments were approved by the independent Institutional Review Boards of relevant institutions listed below. All patients provided informed written consent prior to participating in the studies. Genetic analysis of samples in China was approved by the Administration Office of National Human Genetic Resources of China.

### Ethics committees and institutional review boards

The following ethics committees/institutional review boards, listed by country, considered and approved the study protocol.

**Australia:** Greenslopes Research and Ethics Committee, Greenslopes; St Vincent's Hospital (Melbourne) HREC D, Melbourne; Research Ethics Committee Royal Brisbane Hospital, Herston; CSAHS Human Research Ethics Committee- CRGH Zone, Concord.

**Brazil:** Comitê de Ética e Pesquisa do Hospital das Clínicas e Faculdade de Medicina de Ribeirão Preto da USP–HCFM, São Paulo; Comitê de Ética e Pesquisa da Faculdade de Ciências Médicas—UNICAMP, São Paulo; Comitê de Ética e Pesquisa do Hospital Universitário Prof.Edgard Santos-UFBA, Bahia; Comitê de Ética e Pesquisa da Faculdade de Medicina do ABC\Fundação do ABC—FMABC, São Paulo; Comitê de Ética e Pesquisa do Hospital das Clínicas da Faculdade de Medicina da USP–HCFMUSP, São Paulo; Faculdade de Ciencias Medicas, São Paulo, Bioethiccs Research Committee, Salvador Bahia; HCPA Institutional Review Board.

**Canada:** Biomedical Research Ethics Board, Winnipeg.

**China:** EC of Shanghai Public Health Clinical Center, Shanghai; EC of 3rd affiliated hospital of Sun Yat-sen University, Guangzhou; Ethics committee of Ruijin Hospital, Shanghai; Ethics committee of Xiangya Hospital, Changsha; EC of Beijing Friendship Hospital, Beijing; EC of Beijing You’an Hospital, Beijing; Zhe Jiang University Ethics Committee, Hangzhou; Shanghai Second Medical University Ethics Committee, Shanghai; Jing An Central Hospital Ethics Committee, Shanghai; 1st Hospital of Beijing University Ethics Committee, Beijing; Beijing 302 Hospital Ethics Committee, Beijing; The Ethics Committee of Beijing Ditan Hospital, Beijing; The 1st Hospital of West China University of Medical Science Ethics Committee, Chengdu; Shanghai Hua Shan Hospital Ethics Committee, Shanghai; Shanghai Changhai Hospital Ethics Committee, Shanghai; 2nd Affiliated Hospital of Chongqing Medical University Ethics Committee, Chongqing; Ethics Committee of 1st PLA Medical University, Guangzhou; The EC of 1st affiliated Hospital, College of Medical Science Zhejiang University, Hangzhou; The EC of Shanghai Huashan Hospital, Shanghai; Ethics Committee of People's Hospital, Peking University, Beijing.

**France:** Comité de Protection des Personnes Ile-de-France IV, Paris; Independent Ethics Committee of Paris Saint Louis (CCPPRB Paris Saint Louis), Paris.

**Germany:** Landesamt fuer Gesundheit und Soziales (LaGeSo), Berlin; Ethik-Kommission der Medizinischen Fakultaet der Universitaet zu Koeln, Koeln; Ethik-Kommission der Albert-Ludwigs-Universitaet Freiburg, Freiburg; Ethics Committee of the Campus Virchow Klinikum, Berlin; Ethics Committee of the Medical Faculty Ludwig Maximilians University, Munich; Ethik-Kommission der Medizinische Fakultät, Klinikum der Universität München; UniveHannover Medical University (MHH) Ethics Committee, Hannover; Ethics Committee of the Faculty of Medicine University of Düsseldorf, Düsseldorf; Ethics Committee Ruhr University Bochum, Bochum; Ethik-Kommission der Universitätsklinikum Frankfurt, Frankfurt.

**Greece:** Board of Directors "G. Papanikolaou" Hospital, Thessaloniki; Board of Directors "H. Dynant" Hospital, Athens; Board of Directors University Hospital of Ioannina, Ioannina; Board of Directors "Papageorgiou" Gen. District Hospital of Thessaloniki.

**Hong Kong:** HKU/HA HKW Institutional Review Board, Hong Kong; Joint CUHK-NTEC Clinical Research Ethics Committee, Hong Kong; Clinical & Research EthicsCommittee, KW Cluster, Hong Kong.

**Italy:** Segreteria Comitato Etico, Pisa; Comitato Etico Policlinico Universitario di Cagliari, Cagliari; Comitato Etico Policlinico Universitario di Palermo, Palermo; Comitato Etico Indipendente Locale Azienda Ospedaliera, Bari; Comitato Etico Azienda Ospedaliera, Brescia.

**Israel:** The State of Israel Ministry of Health, Tel Aviv; Rabin Medical Center Affiliated with the Tel Aviv University, Petach-Tiqwa; Rambam Medical Center, Haifa.

**Korea:** Seoul National Univ. Ethics Committee, Seoul; Asan Medical Center Ethics Committee, Seoul; Severance Hospital–Yonsei University Ethics Committee, Seoul; Yonsei University College of Medicine, Seoul; Samsung Medical Centre Institutional Review Board, South Korea; The Catholic University of Korea, Seoul.

**New Zealand:** Northern X Ethics Committee, Auckland; Auckland Ethics Committee, Auckland.

**Poland:** Committee of Bioethics Ludwik Rydygier Memorial Medical University of Bydgoszcz, Bydgoszez; Bioethics Committee of Postgraduate Medical Training Centre, Warsaw; Committee of Bioethics Medical University in Bialystok, Bialystok; Bioethics Committee of Medical University of Lodz, CMKP Bioethics Committee, Warsaw.

**Russia:** Ethics Committee of Stavropol State Medical Academy, Stavropol; Ethics Committee of Hepatologist LLC, Samara.

**Singapore:** Central IRB Domain E, Singapore; Singapore General Hospital Ethics Committee, Singapore; Parkway Independent Ethics Committee, Singapore.

**Spain:** Hospital General Universitario de Valencia Comité Etico de Investigación Clínica, Valencia; Hospital Virgen del Rocio Comité Etico de Investigación, Clínic, Sevilla.

**Switzerland:** Ethics Committee of both Basel EKBB, Basel; Kantonale Ethik-Kommission Bern, Bern;.

**Taiwan:** Ethics Committee National Taiwan University Hospital, Taipei; Joint Institutional Review Board Department of Medical Research Education, Taipei; Institutional Review Board, Taipei Veterans General Hospital, Taipei; The Protection of Human Subjects Institutional Review Board Tri-Service General Hospital, Taipei.

**Thailand:** The Ethics Committee, Prince of Songkla University, Had Yai; Ethical Review Committee Royal Thai Army Medical Department, Bangkok; Ethical Clearance Committee on Human Rights Related to Researches Involving Human Subjects, Bangkok; Research Ethics Committee, Faculty of Medicine, Chiang Mai University, Chiang Mai; Siriraj Institutional Review Board, Bangkok; The Khon Kaen University Ethics Committee For Human Research, Khon Kaen; The Ethics Committee, Faculty of Medicine, Chulalongkorn University, Bangkok.

**Turkey:** Marmara Universitesi Tip Fakultesi Arastirma Etik Kurulu, Istanbul; Ankara Universitesi Tip Fakultesi Etik Kurulu, Ankara; Cukurova Universitesi Tip Fakultesi Etik Kurulu, Adana; Istanbul Universitesi Istanbul Tip Fakultesi Yerel Etik Kurulu, Istanbul; Uludag Universitesi Universitesi Tip Fakultesi Etik Kurulu, Bursa.

**United States:** California Pacific Medical Center Institutional Review Board, San Francisco; McGuire Institutional Review Board, Richmond; Stanford University Research Compliance Office, Stanford; Chesapeake Research Review, Inc., Columbia; Kaiser Permanente Northwest Institutional Review Board, Portland; Office for the Protection of Research Subjects, Los Angeles; Institutional Review Board Ernory University, Atlanta; Mayo Foundation Institutional Review Board, Rochester; Western Institutional Review Board, Olympia; New England Medical Centre, Boston; Committee on the Protection of the Rights of Human Subjects, The School of Medicine, Chapel Hill; California Pacific Medical Center Institutional Review Board, San Francisco; Western Institutional Review Board, Olympia; Human Subjects Review Committee, Seattle; Human Subjects Division, University of Michigan Medical School, Ann Arbor; Cedars-Sinai Medical Center, Office of Research Compliance, Beverly Hills; Minnesota Clinical Research Center, Institutional Human Research Committee, Minneapolis; Institutional Review Board Huntington Memorial Hospital; Pasadena; Institutional Review Board; St Francis Medical Center, Honolulu; Institutional Review Board, University of Puerto Rico Medical Sciences Campus, San Juan, Puerto Rico.

## Results

A total of 701 patients with CHB who received peginterferon alfa-2a 180 μg/week for 48 weeks had *IFNL3* genotype data available and were included in this analysis. Most patients (614, 88%) were Asian. For the majority of analyses patients were divided into four populations: Asian, HBeAg-positive (n = 428), Asian, HBeAg-negative (n = 186), white, HBeAg-positive (n = 31), and white, HBeAg-negative (n = 47). Nine patients who were not classified as either Asian or white were not included in analyses according to race. Among the 465 HBeAg-positive patients, 233 (50%) were infected with HBV genotype C and 125 (27%) with genotype B. Among the 236 HBeAg-negative patients, 96 (41%) were infected with genotype C and 55 (23%) with genotype B. The discontinuation rate in the original three trials was low (5–7%) and few patients were excluded for failure to complete 48 weeks of planned treatment. The number of patients from each of the original three trials is shown in Fig A in S1 File. The baseline characteristics of HBeAg-positive and HBeAg-negative patients are shown in Table 1, stratified by ethnicity. HBV genotypes B and C predominated in Asian patients, whereas genotypes A and D were more common in white patients (Table 1).

**Table 1:** Demographic and clinical characteristics of patients Included in the analyses.

Characteristic	HBeAg-Positive Patients			HBeAg-Negative Patients		
	All patients (n = 465)^a^	Asian patients (n = 428)	White patients (n = 31)	All patients (n = 236)^b^	Asian patients (n = 186)	White patients (n = 47)
**Male, n (%)**	345 (74.2)	315 (73.6)	25 (80.6)	196 (83.1)	158 (84.9)	35 (74.5)
**Female, n (%)**	120 (25.8)	113 (26.4)	6 (19.4)	40 (16.9)	28 (15.1)	12 (25.5)
**Mean ± SD age, years**	32.0 ± 9.6	32.0 ± 9.3	33.2 ± 13.4	38.8 ± 10.8	38.1 ± 10.9	41.0 ± 9.7
**HBV genotype, n (%)**						
**A**	24 (5.2)	2 (0.5)	20 (64.5)	16 (6.8)	0	15 (31.9)
**B**	125 (26.9)	125 (29.2)	0	55 (23.3)	53 (28.5)	1 (2.1)
**C**	233 (50.1)	231 (54.0)	0	96 (40.7)	96 (51.6)	0
**D**	7 (1.5)	0	7 (22.6)	24 (10.2)	0	24 (51.1)
**Other/unknown**	76 (16.3)	70 (16.4)	4 (12.9)	45 (19.1)	37 (19.9)	7 (14.9)
**Mean ± SD HBV DNA level, log**_**10**_	9.5 ± 2.0	9.5 ± 2.0	9.4 ± 2.1	7.2 ± 2.0	7.3 ± 2.1	7.0 ± 1.35
**Mean ± SD ALT level, log**_**10**_	2.0 ± 0.3	2.0 ± 0.3	1.9 ± 0.3	1.8 ± 0.3	1.8 ± 0.3	1.8 ± 0.3
**Response rate, n (%)**	119 (25.6)	109 (25.5)	10 (32.3)	110 (46.6)	97 (52.2)	13 (27.7)
***IFNL3* genotype distribution, n (%)**						
**rs12979860**						
**CC**	353/442 (79.9)	339/405 (83.7)*	11/31 (35.5)	164/209 (78.5)	137/159 (86.2)*	26/47 (55.3)
**non-CC**	89/442 (20.1)	66/405 (16.3)	20/31 (64.5)	45/209 (21.5)	22/159 (13.8)	21/47 (44.7)
**rs12980275**						
**AA**	374/457 (81.8)	359/420 (85.5)*	12/31 (38.7)	189/230 (82.2)	162/180 (90.0)*	26/47 (55.3)
**non-AA**	83/457 (18.2)	61/420 (14.5)	19/31 (61.3)	41/230 (17.8)	18/180 (10.0)	21/47 (44.7)
**rs8099917**						
**TT**	398/457 (87.1)	372/420 (88.6)*	20/31 (64.5)	199/230 (86.5)	164/180 (91.1)*	32/47 (68.1)
**non-TT**	59/457 (12.9)	48/420 (11.4)	11/31 (35.5)	31/230 (13.5)	16/180 (8.9)	15/47 (31.9)

ALT, alanine aminotransferase; HBeAg, hepatitis B e antigen; HBV, hepatitis B virus; SD, standard deviation.

^a^ Includes four black patients, one non-Asian patient, and one Pacific Islander.

^b^ Includes two black patients and one non-Asian patient.

* p<0.0001 (Chi-square) for the difference in genotype distribution between the Asian and white populations.

The genotype distribution of *IFNL3* genotypes at rs12979860 in HBeAg-positive and HBeAg-negative patients is shown in Table 1. The overall frequency of CC and non-CC genotypes was similar in HBeAg-positive and HBeAg-negative patients. In the HBeAg-positive population, the CC genotype was more common in Asian (84%) than in white (36%) patients (p<0.0001). Similarly, in the HBeAg-negative population more Asian than white patients (86% versus 55%, p<0.0001) had a CC genotype. Similar patterns in genotype distribution were observed at rs12980275 and rs8099917 (Table 1). Linkage disequilibrium r^2^ results indicated the strongest linkage disequilibrium was between rs12979860 and rs12980275, with r^2^ values 0.68–1.0 across the subgroups of Asian and white HBeAg-positive and -negative patients. Scores were similar for rs12979860 and rs8099917 (r^2^ 0.43–0.65) and for rs12980275 and rs8099917 (r^2^ 0.47–0.79) (Table A in S1 File). Fisher’s exact p-values, to test for deviation from Hardy–Weinberg equilibrium for rs12980275, rs12979860, and rs8099917, respectively, were 0.296, 0.011, and 0.188 (HBeAg-positive Asian patients), 1.0, 0.212, and 1.0 (HBeAg-negative Asian patients), 0.420, 0.242, and 1.0 (HBeAg-positive white patients) and 0.705, 0.705, and 0.578 (HBeAg-negative white patients). The deviation from Hardy–Weinberg equilibrium for rs12979860 in HBeAg-positive Asian patients could indicate either violation of one or more assumptions of Hardy–Weinberg equilibrium, could be an indication of genotyping errors [29], or could be by chance. The potential consequence of such a deviation is a biased estimate of the association’s magnitude for rs12979860 in HBeAg-positive Asian patients.

The overall response rate was 26% (119/465) among HBeAg-positive patients and 47% (110/236) among HBeAg-negative patients.

**Fig 1:**
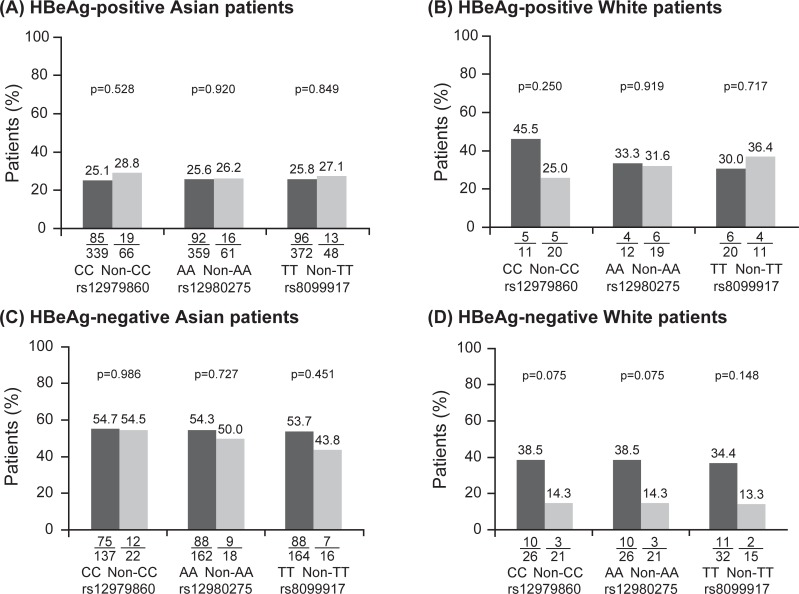
Response rates according to host *IFNL3* genotype. Response rates according to genotype at rs12980275, rs12979860, and rs8099917 in HBeAg-positive Asian (A) and white (B) patients, and in HBeAg-negative Asian (C) and white (D) patients. Some patients did not have data available for all genotypes, and response rates are calculated on the basis of the number of patients with data.

Asian and white patients were analyzed separately, given the difference in frequency of HBV genotypes between the two groups (Table 1). Results of the univariate logistic regression analysis indicated that there were no statistically significant associations between treatment response and genotype at any of the three SNPs in the HBeAg-positive Asian (Fig 1A) or white (Fig 1B) populations, or in the HBeAg-negative Asian (Fig 1C) or white populations (Fig 1D). The results did not change when HBeAg seroconversion alone was considered as a treatment outcome in HBeAg-positive patients (Table 2). In both the HBeAg-positive and -negative populations, numerically lower response rates were observed among white carriers of non-CC rs12979860 genotypes compared with carriers of CC rs12979860 genotypes (Fig 1B and 1D); however, as both analyses included only a very small number of samples, these differences failed to reach statistical significance.

**Table 2:** Rates of HBeAg seroconversion in HBeAg-positive patients according to genotype at rs12980275, rs12979860, and rs8099917. Some patients did not have data available for all genotypes, and percentages are calculated on the basis of the number of patients with data.

HBeAg seroconversion rate, n (%)	*IFNL3* genotype								
	rs12979860			rs12980275			rs8099917		
	CC	non-CC	p-value	AA	non-AA	p-value	TT	non-TT	p-value
**All patients**	124/353 (35.1)	33/89 (37.1)	0.7311	134/374 (35.8)	30/83 (36.1)	0.9567	143/398 (35.9)	20/59 (33.9)	0.7612
**Asian patients**	117/339 (34.5)	25/66 (37.9)	0.6003	127/359 (35.4)	22/61 (36.1)	0.916	133/372 (35.8)	15/48 (31.3)	0.5394
**White patients**	6/11 (54.5)	8/20 (40.0)	0.4383	6/12 (50.0)	8/19 (42.1)	0.6674	9/20 (45.0)	5/11 (45.5)	0.9806

### Exploration of baseline covariates

**Fig 2:**
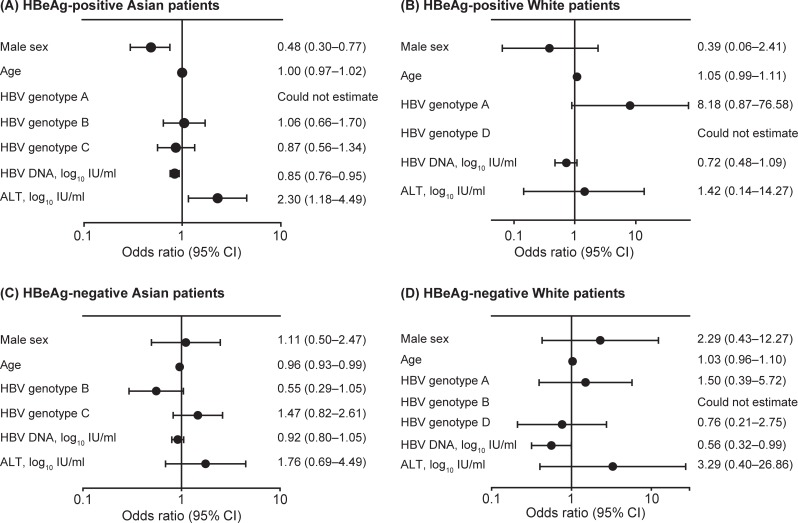
Associations between baseline covariates and treatment outcome in univariate logistic regression analyses. Associations are shown for HBeAg-positive Asian (A) and white (B) patients, and in HBeAg-negative Asian (C) and white (D) patients. ALT, alanine aminotransferase; HBeAg, hepatitis B e antigen; HBV, hepatitis B virus. After adjusting for significant baseline covariates, there were no statistically significant associations between *IFNL3* genotypes and response in the final logistic regression models (Fig 3).

Associations between baseline covariates and treatment response were explored separately by univariate logistic regression analyses in HBeAg-positive Asian and white patients (Fig 2A and 2B) and in HBeAg-negative Asian and white patients (Fig 2C and 2D). For HBeAg-positive Asian patients, significant covariates (p<0.05) were sex, HBV DNA, and ALT. For HBeAg-negative Asian patients, age was the only significant baseline predictor of response. For HBeAg-negative white patients, HBV DNA was found to be significant. No significant associations were found for HBeAg positive white patients.

**Fig 3:**
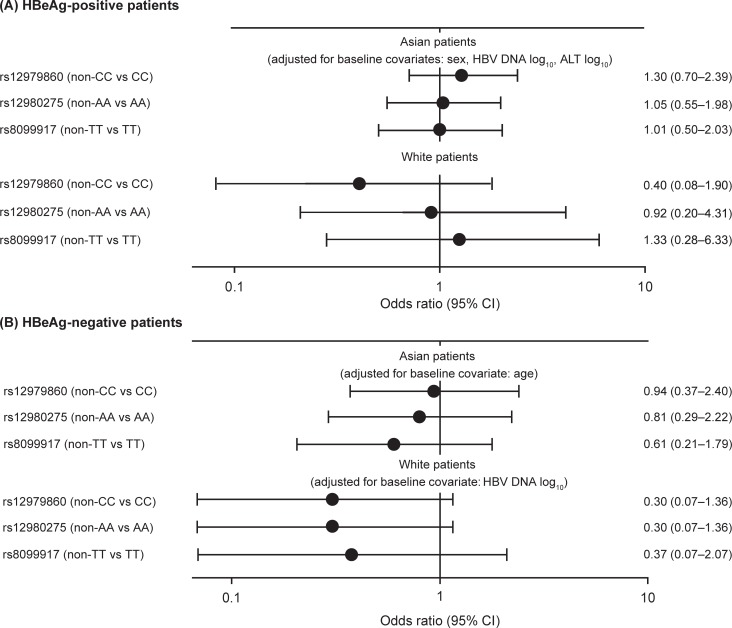
Associations between baseline covariates and treatment outcome in multivariate logistic regression analyses. Associations are shown for HBeAg-positive (A) and HBeAg-negative (B) patients. ALT, alanine aminotransferase; HBeAg, hepatitis B e antigen; HBV, hepatitis B virus.

### HBsAg loss

A total of 18 of 465 (3.9%) HBeAg-positive patients (including 12/428 Asian and 6/31 white patients) and 10 of 236 (4.2%) HBeAg-negative patients (including 8/186 Asian and 2/47 white patients) included in the analysis experienced HBsAg loss. Given these low event rates, it was not possible to test for an association between *IFNL3* genotype and HBsAg loss.

## Discussion

The identification of *IFNL3* genotype as a biomarker predicting response to dual PegIFN alfa/ribavirin therapy for chronic HCV infection was immediately useful in the clinic for identifying patients most likely to respond to therapy. A similar tool for stratifying IFN responsiveness in CHB patients would be extremely useful to predict, at baseline, which patients are most likely to respond to PegIFN alfa.

The underlying hypothesis for this study was that *IFNL3* genotype would predict the outcome of treatment with PegIFN alfa in patients with CHB. However, our primary finding was that there was no association between *IFNL3* genotype and treatment response. *IFNL3* genotype was not associated with treatment response in HBeAg-positive or -negative patients of either Asian or white background.

Previous analyses that have examined relationships between these SNPs and response to IFN in patients with CHB have provided discordant results [15–23]. The largest of these analyses (n = 205) considered the relationship between *IFNL3* genotype (rs12980275 and rs12979860) and treatment outcome in HBeAg-positive patients [18]. A significant association was reported between *IFNL3* genotype and the specified primary outcome, which was HBeAg seroconversion alone. However, the relationship between response and *IFNL3* genotype was no longer significant when a more stringent composite endpoint (i.e. HBeAg seroconversion plus HBV DNA level <2000 IU/ml at 24 weeks post-treatment) was considered [18]. Moreover, Sonneveld *et al*. [18] found that *IFNL3* genotype was not a significant predictor of response to IFN when included in a previously calibrated PegIFN treatment index developed by Buster *et al*. [30] Similarly, *IFNL3* genotype was not found to be associated with treatment response in a number of smaller cohorts of patients with HBeAg-positive CHB [15, 17, 21].

Lampertico *et al*. [22] found the rs12979860 CC genotype to be significantly associated with response to IFN in 101 HBeAg-negative patients with HBV genotype D infection who had received IFN-based therapies. *IFNL3* genotype was associated with the primary response (HBsAg clearance) after a median of 11 years of follow-up. *IFNL3* genotype was also associated with response defined as HBV DNA <2000 IU/ml at 6 months after treatment. It is important to note that the median duration of therapy was 23 months (range 10–48 months) [22]. Contrary to the results of Lampertico *et al*., a number of other analyses have not found a significant relationship between *IFNL3* genotype and response to PegIFN alfa in patients with HBeAg-negative CHB [17, 21], including a number of southern European cohorts, most of whom had HBV genotype D infection [20, 23, 31].

The explanation for these discordant results is most likely related to differences in sample size, patient characteristics (including ethnicity and HBV genotype), and the treatment regimen used. One of the strengths of the current study is the large sample size, as well as the very detailed patient phenotypes available from the PegIFN alfa-2a registration studies. The distribution of genotypes in Asian and white patients was consistent with previous reports of minor allele frequency in these populations [32–37]. The data are clear that, among Asian patients, there is no evidence for clinical utility of *IFNL3* genotyping prior to consideration of PegIFN alfa-based therapy. Similarly, the data do not support a role for *IFNL3* genotyping in white CHB patients prior to PegIFN alfa therapy, although we cannot entirely exclude a weak association with IFN responsiveness in this subgroup, given the smaller number of white patients, particularly with HBeAg-negative disease. In addition, it is also possible that subgroups within the heterogeneous Asian population with different minor allele frequencies may show some association.

The explanation for this difference in the relationship between *IFNL3* genotype and IFN responsiveness between HBV and HCV is not clear. HBV and HCV are both hepatotropic viruses, and both respond to IFN therapy *in vitro* and *in vivo*. However, they have a number of fundamental differences in lifecycle and immune-evasion mechanisms. HBV is a DNA virus that has an episomal reservoir in the nucleus (cccDNA), and reverse transcription largely occurs within cytoplasmic capsids, protected from cytoplasmic RNA-sensing mechanisms. The strong association between intrahepatic IFN-stimulated gene expression and IFN responsiveness that is a feature of HCV infection, and that was recently linked to *IFNL3* genotype, has not been described for HBV. Indeed, this seems to be a specific association between *IFNL3* genotype and HCV, as it is not observed in normal liver [38]. Moreover, treatment with PegIFN alfa-2a has been observed to significantly reduce circulating and intrahepatic antigens in the absence of immune cell responses in a murine model of HBV infection [39]. Thus, fundamental differences in the host–virus relationship for HBV and HCV are clearly present, and remain an important focus for future research.

Prokunina-Olsson *et al* [40] have described a variant upstream of *IFNL3* that creates a novel interferon gene, *IFNL4*, that is linked with HCV clearance in PegIFN-treated patients, possibly more so even than SNPs in *IFNL3*. The role of this variant in HBV response to PegIFN is currently unknown, however, and was not investigated in the current study.

In conclusion, the present study is the largest analysis to date of the association between *IFNL3* genotype and response to IFN in patients with CHB. The data suggest that *IFNL3* polymorphisms are not a major determinant of response to PegIFN alfa-2a in patients with HBeAg-positive or HBeAg-negative CHB. When selecting patients for treatment with PegIFN alfa-2a, clinicians should continue to use established baseline predictors of response.

## Supporting information

S1 File Patients included from the original studies (Fig A) and Linkage disequilibrium r2 values for each SNP combination in different subgroups (Table A).
(DOCX)
