# Supplementary material for: No association between IFNL3 (IL28B) genotype and response to peginterferon alfa-2a in HBeAg-positive or -negative chronic hepatitis B
Source: PLoS One. 2018 Jul 17;13(7):e0199198. doi: 10.1371/journal.pone.0199198 (PMC6049926; doi:10.1371/journal.pone.0199198)
Supplement: S1 File — Patients included from the original studies (Fig A) and Linkage disequilibrium r2 values for each SNP combination in different subgroups (Table A). (DOCX) [file pone.0199198.s001.docx]

# Supporting Information

**Fig. A.** **Patients included from the original studies.**


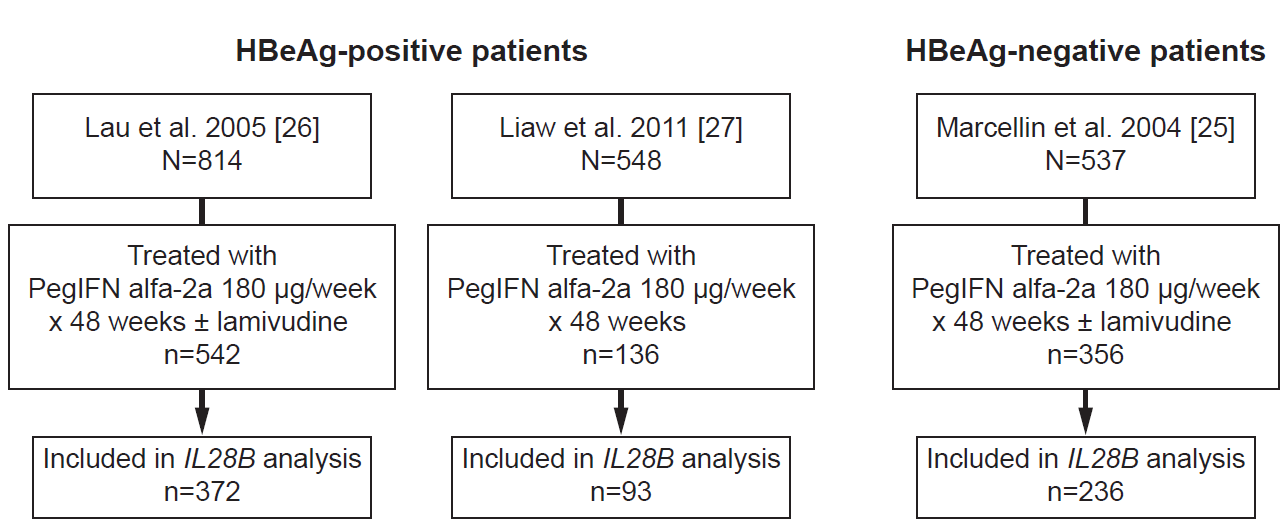


**Table A. Linkage disequilibrium r^2^ values for each SNP combination in different subgroups**

| **Subgroup** | **SNP1** | **SNP2** | **r^2^** |
| --- | --- | --- | --- |
| **HBeAg-Positive Asian** | rs12980275 | rs12979860 | 0.72 |
|  | rs12980275 | rs8099917 | 0.79 |
|  | rs12979860 | rs8099917 | 0.65 |
| **HBeAg-Negative Asian** | rs12980275 | rs12979860 | 0.68 |
|  | rs12980275 | rs8099917 | 0.76 |
|  | rs12979860 | rs8099917 | 0.61 |
| **HBeAg-Positive White** | rs12980275 | rs12979860 | 0.74 |
|  | rs12980275 | rs8099917 | 0.47 |
|  | rs12979860 | rs8099917 | 0.43 |
| **HBeAg-Negative White** | rs12980275 | rs12979860 | 1 |
|  | rs12980275 | rs8099917 | 0.58 |
|  | rs12979860 | rs8099917 | 0.58 |
